# Evaluation of Benzene Exposure and Early Biomarkers of Kidney Damage in Children Exposed to Solvents Due to Precarious Work in Ticul, Yucatán, México

**DOI:** 10.5334/aogh.2482

**Published:** 2019-07-03

**Authors:** Norma Pérez-Herrera, Lorena Díaz de León-Martínez, Rogelio Flores-Ramírez, Olivier Barbier, Manolo Ortega-Romero, Fernando May-Euán, Kelvin Saldaña-Villanueva, Javier Perera-Rios, Francisco Javier Pérez-Vázquez

**Affiliations:** 1 Coordinación para la Innovación y Aplicación de la Ciencia y la Tecnología (CIACYT), Universidad Autónoma de San Luis Potosí, San Luis Potosí, MX; 2 Centro de Investigación Aplicada en Ambiente y Salud (CIAAS), Coordinación para la Innovación y Aplicación de la Ciencia y la Tecnología (CIACYT), Universidad Autónoma de San Luis Potosí, San Luis Potosí, MX; 3 Facultad de Medicina, Universidad Autónoma de Yucatán, Mérida, Yucatán, MX; 4 Departamento de Toxicología, Centro de Investigación y de Estudios Avanzados del Instituto Politécnico Nacional (CINVESTAV-IPN), México, Ciudad de México, MX

## Abstract

**Background:**

The child labor situation has been associated with precarious job conditions and poor health conditions because children are often exposed to unsafe work environments, stressful psycho-social work conditions, scarce or no access to protective services, and heavy work burdens.

**Objective:**

The aim of the study was to evaluate markers of exposure to benzene through the exposure biomarker trans, trans-muconic acid (tt-MA), and biomarkers of early renal damage in children who work in sites that are under precarious job conditions.

**Method:**

Samples of urine were obtained from children (aged 6–12 years old) who resided in Ticul, Yucatan, Mexico. Exposure to benzene was assessed through trans, trans-muconic acid (t,t-MA). Evaluated renal damage biomarkers were: Cystatin-C (Cys-C), Osteopontin (OPN), α1-Microglobulin (α1-MG) and Neutrophil Gelatinase-Associated Lipocalin (NGAL).

**Findings:**

Children who live where the workplace is inside the dwelling presented higher mean levels of tt-MA (0.59 mg/g creatinine) compared with those who live away from the workshops (0.19 mg/g creatinine). Likewise, mean levels of NGAL (4.7, 5.2 ng/ml), albuminuria (10, 10 ng/ml), Cys-C (11.8, 7.5 ng/ml), OPN (224.4, 226.5 ng/ml) and α1-MG (96.6, 73.6 ng/ml) were found in children where the workplace was inside the dwelling and outside, respectively.

**Conclusion:**

Our data indicate that the children who work under precarious job conditions are exposed to benzene, and they exhibit protein levels that suggest renal damage in a population in precarious working conditions. Therefore, the child population should be considered as the most vulnerable and susceptible to suffer adverse health effects.

## Introduction

The International Labor Organization states that currently 218 million children between 5 and 17 years old are engaged in economic production, of whom 152 million are participants in child labor; almost fifty percent of the children are in a precarious work situation. Child labor is concentrated in activities such as agriculture (71%), services (17%), and the industry sector, particularly mining (12%) [1].

In Mexico, approximately 3.2 million children aged 5 to 17 years carry out an economic activity, of which 88% perform activities that represent high health risks [2]. The child labor situation has been associated with precarious work and poor health conditions because children are often exposed to hazardous environments, stressful psycho-social work conditions, scarce or no access to protective services, and heavy work burdens [3]. In addition, one of the characteristics of precarious child labor is the lack of access to health services. The seriousness also depends on the places where the activities are carried out; this kind of population is located in communities or neighborhoods, where the work is located in the dwelling itself [4].

Footwear manufacturing is an activity where children’s work takes place in an important manner [5] and working conditions are reported to be dangerous [6]. According to several studies, children participating in the process are responsible for gluing the soles of the shoes or sewing the pieces [5]. One of the biggest concerns of this activity is the exposure to organic compounds that result from the use of adhesives and paints, such as benzene, ethylbenzene, toluene, and xylene [7]. In this context, long exposure to this chemical produces adverse effects on bone marrow and has been associated with anemia and leukemia [8,9]. In addition, epidemiological and experimental studies have shown that this can cause non-cancerous alterations to the renal immune system as well as alterations such as tubular acidosis, hypokalemia, azotemia, hematuria, and proteinuria [10,11]. Therefore, assessment of benzene exposure must be done in order to characterize the risk in areas with potential air pollution.

Footwear manufacturing can be conceptualized as a family nuclei of hazardous job conditions due to the inclusion of practically the whole family. However, children disproportionately suffer adverse health effects due to their relationship with the environment during their development; in this period the child population is markedly different from adults’ due to their behavior and biology [12]. There is evidence of environmental stressors, exposures to toxic substances, and certain environments that specifically affect children’s health and development throughout life [13,14,15,16].

Consequently, child labor becomes a public health problem that not only affects families by increasing their economic vulnerability, but also has an impact because of the magnitude in the stability of health services, due to the need to care for an increase in non-communicable diseases (many contaminants affect the nervous system, cardiovascular system, kidneys, lungs, endocrine system, among others).

The difficulties of predicting diseases such those affecting the renal system constitutes a heterogeneous spectrum of etiologies, pathological stages, and genetic bases. Therefore, the need arises to identify new biomarkers to establish health surveillance for the timely detection of this disease. One of the limitations for the renal function assessment is the use of non-sensitive and non-specific biomarkers, such as serum creatinine (SCr) [17]. In this regard, Kidney Injury Molecule 1 (KIM-1), Neutrophil Gelatinase-Associates Lipocalin (NGAL), and Cystatin C (Cys-C) have been shown to be more sensitive than SCr and can detect specific tubular and glomerular damage [18,19].

Thus, strategies for the timely detection of kidney disease should be implemented in order to avoid or delay the possible damage that these may cause. The use of biomarkers as informative tools for the health assessment has increased in occupational health because they can be used to reveal occupational exposure and environmental impact in communities by classifying and quantifying the exposure related to work environments [20]. The main objective of the study was to highlight the problems presented by child population in hazardous job conditions of working in footwear manufacturing, evaluating exposure to benzene and renal damage.

## Methods

### Study Population

The study area was the municipality of Ticul located in the south of the state of Yucatan, Mexico. According to data from the National Council for the Evaluation of Social Development Policy (CONEVAL), 61% of the population of Ticul lives in poverty [21] and more than 50% is of indigenous origin, reported by the National Population Council [22]. The National Institute of Geography and Statistics mention that the main economic activity in this community is the footwear manufacturing industry, with an estimated production of 453,600 pairs of shoes per year from 135 shoemaker workshops [23]. The child population from 6 to 12 years old was evaluated through convenience sampling in footwear manufacturing, agricultural, and municipal garbage dump areas. A total of 41 children who met the inclusion criteria were included. One child per house was assessed. Forty-four percent of the participating children lived in houses where a footwear manufacturing workshop was located. A questionnaire to record age, sex, work, and workplace, among other population characteristics and health habits, was given by trained staff. Height was measured with a stadiometer; weight and body mass index (BMI) were obtained by means of a bioelectric impedance scale (Tanita BC-601F, USA). The research methodology was carried out with the approval of the Bioethics Committee of the Faculty of Medicine from the Autonomous University of Yucatan (UADY).

### Urine sample collection

Urine samples were obtained from the first-morning micturition and were collected in sterile 50 mL bottles. The samples were immediately stored at –20°C for preservation.

### Urinary trans, trans-muconic acid determination

Trans, trans-muconic acid (tt-MA) in urine has been used as a biomarker of benzene exposure. However, is necessary to consider that tt-MA is also a metabolite of sorbic acid. In this regard, the consumption of food and drinks such as green tea, potato chips, bread, coffee, and wine or smoking can result in an overestimation of urinary tt-MA and HA concentrations and cause false-positive results during biological monitoring. Therefore, one of the main indications was not to consume the aforementioned products two days before the sampling. The determination of tt-MA was performed based on the method described by Ducos et al. [24] The urine samples were centrifuged at 2000 rpm for 15 min to separate the suspended material and filtered on a 0.22 μm Millipore pore size filter to remove suspended particles. One milliliter of urine was treated with 2.0 mL of Trizma Buffer (pH 8.5); later an extraction was performed with SAX cartridges previously conditioned with methanol, distilled water, and a buffer solution. The cartridges were washed with a methanol/acetic acid solution (99: 1) and eluted with methanol/acetic acid (80: 20). The solution was filtered through a Millipore 0.45 μm pore size filter and transferred to a salinized vial. Quantification was performed on a high-performance liquid chromatograph (Agilent Technologies 1260) coupled with a diode array detector. The limit of detection was 0.03 mg/l and as quality control, an IRIS ClinCal Recipe 9969 standard was used (Munich, Germany). The recovery percentage was 98%. The urinary concentrations of tt-MA were adjusted using urinary creatinine levels determined from the Jaffe colorimetric method [25].

### Renal damage biomarkers assessment

Cystatin-C (Cys-C), Osteopontin (OPN), α1-Microglobulin (α1-MG), and Neutrophil Gelatinase-Associated Lipocalin (NGAL) were evaluated using a custom human magnetic luminex® screening assay (R&D Systems, Inc., Minneapolis MN, USA), and concentrations of renal biomarkers were obtained by means of a Luminex xMAP® Instrument (MAGPIX®, Luminex Corp., Austin TX, USA). The measures were performed following the manufacturer’s instructions and based on the methodology described by Diaz de Leon et al. [26] All samples were analyzed in duplicates and the intra-assay CV was below 15%.

### Statistics

The levels of tt-MA and renal disease biomarkers were compared between children where the workplace was inside the dwelling and outside the dwelling using the Mann–Whitney *U* test to compare. We used JMP IN Start Statistics Software 5.0 (North Carolina, USA) for all statistical analyses.

## Results

Ticul is an indigenous community with 37,685 inhabitants, whose child population between 6 and 12 years old is 5,601 and concentrates their school attendance in eight schools [23]. In this community, population is mainly engaged in footwear manufacturing, reflecting the family’s precariousness, because all members of the family, including children, are usually involved in different activities for the development of footwear. Table 1 shows the characteristics of the children evaluated in this study. A total of 41 children dedicated to shoe manufacturing processes were evaluated, with an average age of 8.5 ± 2.5 years with weight and height of 30.8 ± 9.8 kg and 129.4 ± 12.5 cm, respectively. Regarding BMI, only 6% of children participating in the study were classified as overweight and 65% were underweight. Also, the results showed that 12% of the children presented chronic undernutrition according to the height-for-age scores (H/A Z-scores < –2) based on the growth standards of the World Health Organization (WHO) [27]. Moreover, 3% of children presented chronic severe undernutrition. Acute undernutrition (W/A z-scores < –2) occurred in 9% of the population and 33% were classified as overweight. On the other hand, 100% of the visited workshops (n = 10) lacked security measures (no mask or gloves, inappropriate clothing, etc., which compromises the inhalation route of exposure to benzene). Additionally, children spent more than four hours in these places (data not shown).

**Table 1:** Descriptive characteristics of children from Ticul.

Characteristics	

Children (N)	41
Boys (%)	44
Age (years)	8.5 ± 2.5
Height (cm)	129.4 ± 12.5
Weight (Kg)	30.8 ± 9.8
BMI (Kg/m^2^) (%)	
Underweight	65
Normal weight	29
Overweight	6
Height for age (Z-Score H/A)^#^ %	
Moderate chronic undernutrition (<–2)	9
Severe chronic undernutrition (<–3)	3
Weight for age (Z-Score W/A)^#^ %	
Overweight (>1)	33
Acute moderate malnutrition (<–2)	9
Child burden* (%)	15
Home/workplace° (%)	44
Drinking water (%)	20

^#^ Calculated by Z ranges of child growth standards proposed by the World Health Organization (WHO). * Children who study and work. ° Children whose workplace is inside the house they inhabit.

These scenarios are recurrent in children in a hazardous environment. In addition, in these activities, there are greater health risks due to low nutritional status. Also, the workplaces are usually located in the dwelling; therefore, they can be exposed to different chemical compounds used in footwear manufacturing. For example, the children who participated in the assembly of the shoe, which consists of shaping and adding ribbons or ornaments, are constantly involved in the use of adhesives. Likewise, the finishing of the shoe is one of the activities carried out by children, utilizing paints, cleaning with solvents, gasoline, and lacquer; therefore, children may be exposed to compounds released by these substances.

Table 2 shows the concentration of urinary tt-MA found. It was observed that children who lived where the workplace was inside the dwelling presented median urinary t-t MA levels (0.59 mg/g creatinine) higher compared to those who lived outside the workshop (0.20 mg/g creatinine); although there was no difference between the two groups, there was a greater variation in those who lived where the workshop was inside the dwelling than the ones who outside the workshop (1.09–0.38 mg/g creatinine, respectively). In the same way, the percentage of children over the biological exposure index (>0.5 mg/g Cr) [28] was higher in children where the workplace was inside the dwelling (28%) than outside the dwelling (17%).

**Table 2:** Levels of benzene exposure.

Workplace	Mean	SD	P25	P50	P75	BEI^&^

**Inside (N = 18)**	0.59	1.09	<LOD	0.06	0.67	28
**Outside (N = 23)**	0.20	0.38	<LOD	0.00003	0.23	17

Units expressed in mg tt-MA/g creatinine. ^&^ BEI; Biological exposure index (0.5 mg/g creatinine), expressed as percentage. * Limit of detection (LOD) for tt-MA was approximately 0.05 μ g/L. SD (Standar Deviation), P25, P50 and P75 (percentile).

On the other hand, renal function was assessed by quantifying biomarkers of renal damage (Table 3). The results indicate similar medians of NGAL in children who lived where the workshop was located within the dwelling (4.7 ng/ml) compared to those who lived outside the workshop (5.2 ng/ml). For albuminuria (10, 10 ng/ml), Cys-C (11.8, 7.5 ng/ml), OPN (224.4, 226.5 ng/ml), and α1-MG (96.6, 73.6 ng/ml) were observed in children (workshop inside the house and outside the house, respectively).

**Table 3:** Urinary levels of renal damage biomarkers of Ticul children.

	Inside (N = 18)					Outside (N = 23)				

Parameters	Mean	SD	P25	P50	P75	Mean	SD	P25	P50	P75

NGAL**	12.9	25.1	3.0	4.7	11.7	10.1	11.5	2.9	5.2	12.5
Albuminuria*	25.6	32.6	10.0	10.0	30.0	13.5	7.8	10.0	10.0	10.0
Cys-C**	12.9	10.9	3.7	11.8	19.5	9.2	6.4	3.9	7.5	13.6
OPN**	213.7	126.0	95.5	224.4	331.4	227.7	114.6	166.8	226.5	295.3
α1-MG**	115.2	65.6	63.6	96.6	176.0	95.2	58.2	50.5	73.6	144.9

NGAL: Neutrophil gelatinase-associated lipocalin. Cys-C: cystatin-C. OPN: Osteopontin. a1-MG: α 1-Microglobulin. Mean, SD (Standar Deviation), P25, P50 and P75 (percentile) as: * mg/l and ** ng/ml.

## Discussion

Currently in Mexico the labor and income conditions have shown a great deterioration, as a result of the difficulties in creating quality jobs; the population generates its own employment through the implementation of micro-industries. However, they may be temporary, lacking social benefits, salary reduction, and dangerous job conditions [29]. This makes workers more vulnerable, and the scenario is more worrying due to the inclusion of the entire family, particularly the children.

Ticul is an example of the precarious work that is found in Mexico. Footwear manufacturing processes have been carried out for years in an artisanal way, involving precarious working conditions, such as scarce or no access to health services, without protection, among other things. Likewise, the whole family, including children, has been involved in this activity.

Children who participated in the study showed an overweight percentage below that reported for the national average in rural communities (16.5%) [30]. Also, the children who presented chronic malnutrition according to the height-for-age scores showed values below the percentage of the national average (16.9%) in rural communities of Mexico [31].

Children who are engaged in these activities have greater health risks due to low nutritional status, diseases associated with the exposure to environmental chemical compounds, and a higher prevalence of injuries [32,33]. Families that have a footwear manufacturing workshop inside their dwelling are constantly exposed to solvents through inhalation. In this context, it has been found that people who are engaged in this activity and who use adhesives or paint in the process of manufacturing footwear are exposed to benzene, xylene, ethylbenzene, toluene, and n-hexane [6].

Our results show that children are exposed to benzene, through the determination of urinary tt-MA. Several studies suggest tt-MA as an exposure biomarker for evaluating human exposure to low levels of environmental benzene. However, when assessing urinary tt-MA as an exposure biomarker in a non-occupationally exposed population (as evaluated in this study), it is necessary to consider that tt-MA is also a metabolite of ascorbic acid. For this reason, it was indicated that the children shouldn’t eat foods such as potato chips, bread, and industrialized juices two days before biological sampling. Our dates of tt-MA are lower than other studies in different exposure scenarios. For example, in an area of Nuevo Leon, Mexico, where an oil refinery is located, higher levels of tt-MA (0.50–1.9 mg/g Creatinine) were found in children from 6 to 15 years old [34]. Moreover, a different scenario regarding the combustion of biomass as a source of exposure that studied children living in indigenous areas of communities from San Luis Potosi, Mexico showed higher values of tt-MA than those found in the present work, with a median of 0.47 mg/g Creatinine, respectively [35]. On the other hand, it has been reported that 70–80% of a child population living in a petrochemical area of the state of Veracruz, Mexico, has lower levels of tt-MA (0.36–0.38 mg/g Creatinine) than the levels found in this study [36]. Other studies of children from marginalized urban areas also showed lower median values of tt-MA (0.220–0.429 mg/g Creatinine [37]. Clearly, these differences are mainly due to the exposure scenario.

Additionally, lower concentrations of benzene have been associated with various toxic effects. For example, DNA oxidative damage (-oxo-7,8-dihydro-2’-deoxyguanosine) has been associated with mean levels of 0.048 mg/g creatinine tt-MA [38]. Also, several studies indicate that exposure to benzene is significantly associated with hematological abnormalities, liver enzyme disorder, and leukemia in children [36,39,40].

We have pointed out that children who live in a hazardous work environment are exposed to chemical substances. In addition, one of the main organs that can be damaged is the kidney. This is of great relevance because the state of Yucatan has increased the prevalence of urolithiasis, where different studies have been associated with extreme hardness of water used for human consumption [41]. In this regard, 20% of the population of this study consumes tap water, representing an environmental risk for the child population, so there is a current need for the implementation of a risk communication program to avoid tap water consumption.

Due to the fact that dangerous labor in Mexico is broad and encompasses millions of people, this is a public health problem for our country. However, there is a consensus on cumulative lifetime environmental exposure in conjunction with other comorbidities, accelerating the rate of deterioration of renal function which increases the risk of kidney disease [42]. In our country, diabetes mellitus, hypertension, and obesity are the most common chronic diseases and are the main risk factors for generating chronic kidney disease [43,44].

In this study, we evaluated proteins that indicate early renal damage. With respect to NGAL, this is a protein of the lipocalin family that has been observed to be increased in chronic renal diseases and acute renal damage; it has also been implemented as a biomarker of early effect to renal damage in children and adults [45]. Mean levels of 9.92 ng/ml and 143.10 ng/ml in boys and girls who lived in areas with presence of nephrotoxic pollutants in drinking water have been reported. The author reported a higher difference between both sexes in NGAL concentrations, possibly caused by pyuria found in girls; [19] nevertheless, in this study only a slight increase was observed in girls compared to boys (data not shown). Cys-C is a protein from the family of cysteine protease inhibitors; several studies have shown that it is an indicator of renal function [46]. In this context, some authors have reported differences in Cys-C concentrations between those who have kidney damage and healthy subjects [47]. Askenazi et al. detected higher median Cys-C levels of 3889 ng/ml in children suffering from kidney damage compared to 2150 ng/ml of Cys-C in healthy children [48]. Concentrations of 590 ng/ml have also been detected in adults with acute kidney damage, which increased with the severity of the disease [49]. The concentrations found in our study were much lower; however, it should be noted that this was assessed in children who did not present any apparent renal damage. In this regard, the serum level of this protein rises earlier than its urinary level and, in order for the Cys-C urine concentration to rise, tubular injury should occur [50]. The urine levels of Cys-C shown in this study was low; therefore there may not present tubular injury.

Osteopontin is a cytokine that is produced by a variety of tissues including the kidney. Evidence shows that urinary OPN concentration was higher in low birth weight infants with acute kidney injury (468 ng/ml) compared with children without acute kidney injury (217 ng/ml), suggesting the utility of the OPN as a predictive biomarker of kidney injury. Considering the previous, our results show similar OPN concentrations to infants without kidney injury. However, several studies have shown that at this level of mRNA, the OPN increase with respect to age [51].

Regarding α1-MG, it is a glycoprotein produced by the liver, which under normal conditions is reabsorbed by proximal tubular cells; [52] therefore any alteration of the proximal tubular cells could increase the amount of α1-MG in urine [53]. Some authors have found it increased in renal damage; in children with idiopathic nephrotic syndrome, it has been found with median values of 2000 ng/ml [54]. In our study, levels are below those previously reported.

It is important to note that there are no cohort values established of these proteins; however, although the values obtained showed that only OPN and NGAL are higher compared to some reports, the presence of proteins prove that there is renal damage.

Our study has presented some limitations, especially the lack of data on environmental concentrations of benzene (as it is done in occupational health); the biomarker of exposure is for an occupational adult scenario and therefore it must be lower in children; and the lack of cohort points in proteins of early renal damage. Nevertheless, children that participated on the study could present risk of renal damage, thus the precautionary principle must be applied. Finally, no other nephrotoxics were evaluated. Also, the evaluation of the exposure biomarker was measured in a spontaneous sample, so in subsequent studies it will be necessary to perform a biomonitoring in the area to establish with greater certainty the exposure values.

It is important to note, that at no time did the current work aim to make the sample representative of the community, since it only sought to define the level of exposure to benzene, the percentage of affected population in kidney health, and the implementation of intervention strategies in working families in hazardous environments (families in Ticul). In this context, these communities could present higher health risks; therefore, they could be more susceptible to toxic effects. It is important to develop interventions to reduce pollutants based on the precautionary principle [55], including benzene, and control the main source.

This is of great relevance because the state of Yucatan has increased the prevalence of urolithiasis, where different studies have been associated with extreme hardness of water used for human consumption [41]. In this regard, 20% of the population of this study consumes tap water, representing an environmental risk for the child population, so there is a current need for the implementation of a risk communication program to avoid tap water consumption.

The new scheme proposed by the World Health Organization through the world conference on primary health care of Astana points to health as a human right [56]. In this sense, notable progress in the health coverage is recognized; however, staying healthy is a challenge, especially for the poor and vulnerable. Efforts to solve problems of health care in the population are dedicated to the treatment of non-communicable diseases (e.g., diabetes mellitus, and hypertension) and in the analysis of the main causal factors such as biological causes (obesity, sex, age, etc.) and behavioral causes (alcoholism, sedentary lifestyle, smoking, eating habits, etc.). In this context, the Astana conference considers acting on social factors such as violence, war, and poverty and environmental factors such as climate change and exposure to toxic substances for the first time. Nevertheless, the context of precarious work has not been considered. Given this new scenario in primary health care, the child population should be considered to be the most vulnerable and susceptible to suffer adverse health effects. Therefore, the risk and exposure biomarkers should be included to monitor exposure to environmental compounds and health in precarious family nuclei and prevent the generation of this kind of diseases.

## Conclusions

Our data indicate that children who work under job insecurity conditions are exposed to benzene, and they present levels proteins that prove renal damage in populations with precarious working conditions according to the OIT. Therefore, the child population should be considered as the most vulnerable and susceptible to suffering adverse health effects.
